# Sample attrition analysis in a prospective cohort study of medical graduates in China

**DOI:** 10.1186/s12874-021-01498-1

**Published:** 2022-01-14

**Authors:** Mingyue Li, Ziyue Wang, Baisong Zhang, Tiantian Wei, Dan Hu, Xiaoyun Liu

**Affiliations:** 1 Department of Health Policy and Management, School of Public Health, Peking University, Beijing, China; 2 China Center for Health Development Studies, Peking University, Beijing, China

**Keywords:** Attrition, Cohort study, Medical graduates

## Abstract

**Background:**

A major challenge of prospective cohort studies is attrition in follow-up surveys. This study investigated attrition in a prospective cohort comprised of medical graduates in China. We described status of attrition, identified participants with higher possibility of attrition, and examined if attrition affect the estimation of the key outcome measures.

**Methods:**

The cohort study recruited 3,620 new medical graduates from four medical universities in central and western China between 2015 and 2019. Online follow-up surveys were conducted on an annual basis. Follow-up status was defined as complete (meaning that the participant completed all the follow-up surveys) and incomplete, while incomplete follow-up was further divided into ‘always-out’, ‘rejoin’ and ‘other’. Multivariable logistic and linear regressions were used to examine factors predicting attrition and the influence on the outcome measures of career development.

**Results:**

2364 (65.3%) participants completed all follow-up surveys. For those with incomplete follow-up, 520 (14.4%) were ‘always-out’, 276 (7.6%) rejoined in the 2020 survey. Willingness to participate in residency training (OR=0.80, 95%CI[0.66 - 0.98]) and willingness to provide sensitive information in the baseline survey predicted a lower rate of attrition (providing scores for university entrance exam OR=0.82, 95%CI[0.69 - 0.97]]; providing contact information (OR=0.46, 95%CI[0.32 - 0.66]); providing household income (OR=0.60, 95%CI[0.43 - 0.84]). Participants with compulsory rural service (OR=1.52, 95%CI[1.05 - 2.19]) and those providing university entrance scores (OR=1.64, 95%CI[1.15-2.33)) were more likely to rejoin in the follow-up survey. These factors associated with follow-up status did not have significant impact on key outcome measures of career development.

**Conclusions:**

Graduates who were unwilling to participate in residency training or not providing sensitive information should be targeted early in the cohort study to reduce attrition. More information about the study should be provided to those graduates early to facilitate their understanding of the meaning in participation. On the contrary, medical graduates with compulsory rural service and those who provided university entrance scores were more likely to rejoin in the cohort. The research team should invest more effort in contacting those graduates and returned them to the cohort.

**Supplementary Information:**

The online version contains supplementary material available at 10.1186/s12874-021-01498-1.

## Background

Longitudinal medical graduates’ cohort studies are invaluable for investigating workforce participation, career development, and mobility of young health professionals, providing insights into the dynamics of the health labor market, and providing guidance for policy-making related to human resources for health [1, 2]. However, one of the key challenges of prospective cohort studies is participant attrition. Attrition is often cumulative so that the non-responders can be a significant proportion of the original sample after a few follow-up surveys, reducing the sample size and thus decreasing the power of estimation [3]. The attrition might happen selectively, and reduce the representativeness of the sample over time and introduce bias [4].

Compared with surveys of general population in social and political sciences or patients in epidemiology, health workers tend to have a lower response rate [5, 6]. For example, in the prospective cohort study of “Medicine in Australia: balancing employment and life (MABEL)” [7, 8], after three follow-up surveys, 34.6% of the GPs and 33.2% of the specialists were lost-to-follow-up due to attrition [3]. In addition, the response rates to surveys of health professionals are found to be declining over time, from over 80% in the 1950 s to less than 50% now [6].

To understand the facilitators of attrition of cohort study in general population, previous studies have found that men, unmarried, current smokers, low socioeconomic status, and poorer health can predict a higher possibility of lost-to-follow-up [9–11]. Young people are also less likely to continuously stay in the cohort compared to older people [11–13]. However, a limited body of research has examined attrition in prospective cohorts specific to young health professionals or young medical graduates, although younger age and health workers are both related to the higher possibility of attrition.

The Cohort Study of Medical Graduates with Compulsory Services in Rural Areas Study was launched in 2015 in four major medical universities from three provinces in China. It is a prospective cohort study comprised of young medical graduates who have just finished their undergraduate medical education. This study was initiated to examine the Compulsory Services Program (CSP) launched in 2010 by the Chinese government [14], which aimed to provide well-trained general practitioners (GP) for rural and remote areas. According to this policy, CSP students do not need to pay for tuition or accommodation during five-year undergraduate study and can receive monthly allowances. In exchange, they need to sign contracts with local health administrations and medical universities on matriculation and commit to going to the appointed township health centers or village clinics to practice for six years after graduation. Maintaining response rate is one of the key challenges in our follow-up surveys, with the concern that sample attrition might introduce considerable bias into the results and conclusions of the cohort study.

In this article, we analyzed the data from this prospective cohort study to investigate the following three questions: (1) what is the status of attrition in a prospective cohort comprised of young medical graduates? (2) in such a cohort, which type of participants are prone to attrition? (3) to what extent does the attrition affect the estimation for key outcome measure of the cohort study? The findings of this paper can provide useful reference for other cohort studies with health professionals to deal with attrition issues.

## Methods

### Study design and data collection

The Cohort Study of Medical Graduates with Compulsory Services in Rural Areas Study is a prospective cohort that investigates the medical study, residency training, employment, and career development of medical graduates, in order to contribute to the development of health workforce in rural and remote areas in China. The study was approved by the institutional review board (IRB) at Peking University Health Science Center (IRB00001052-15027). All participants provided informed consent. In 2015, the first batch of medical graduates trained by CSP graduated after receiving five-year undergraduate study, so we established the first sub-cohort and collected baseline survey at four medical universities since then [15–17]. All CSP classes in the four universities were included in the cohort, and the participation rate was 100% at recruitment. CSP classes were matched with corresponding same-year NCSP classes at 1:1 ratio. We continued establishing sub-cohorts among graduates of 2016, 2017, 2018 and 2019, and conducted follow-up surveys annually. The study included 3,620 medical graduates from the Medical College of Qinghai University (Qinghai province), Jiujiang University (Jiangxi province), Gannan Medical University (Jiangxi province), and Guangxi Medical University (Guangxi Zhuang autonomous region). Participants completed a paper questionnaire at baseline before they finished the undergraduate study. In the baseline survey, we created a WeChat group to include all participants within each school and each sub-cohort (WeChat is a widely used instant messaging app in China. The WeChat group enables to exchange communication between investigators and participants). The links of online self-reported questionnaires were sent each year with notification via WeChat groups, text messages of mobile phones, and E-mails. Till now, we have collected follow-up data in 2016, 2017, 2018, and 2020.

**Fig. 1:**
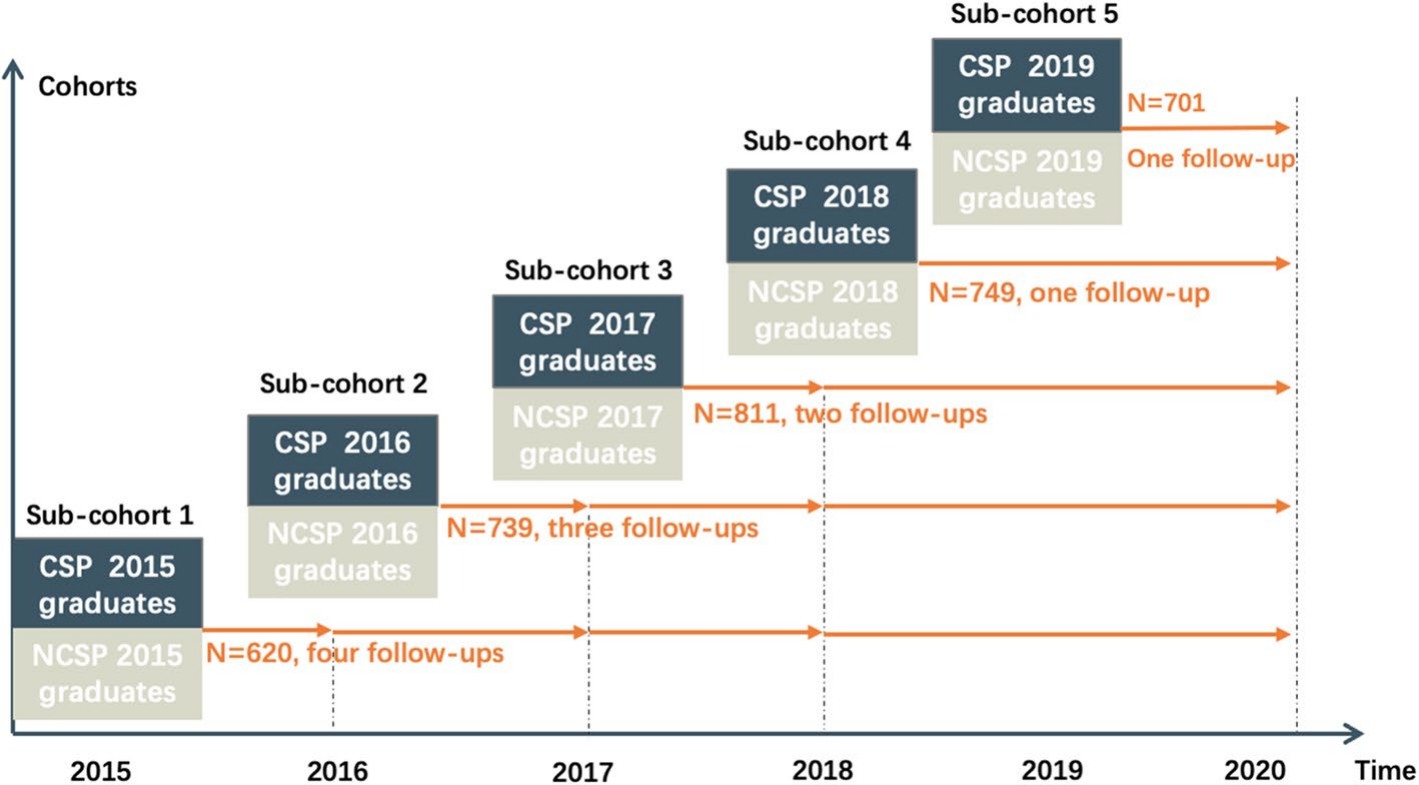
Flow diagram of the Cohort Study of Medical Graduates with Compulsory Services in Rural Areas

Notes: the 2019 follow-up survey was not conducted for graduates of 2015-2018 due to logistic reasons.

There are two types of medical graduates in this study, those who are required to practice in rural and remote areas after graduation (compulsory services program, CSP) and common medical graduates (non-compulsory services program, NCSP). Baseline survey covered demographic characteristics, attitudes towards medical study, and preferences of career development for the two types of graduates. Response behaviors of whether participants provided some sensitive information were also recorded. A flow diagram that summarizes the process, baseline sample size, and follow-ups is presented in Fig. 1.

In the follow-up survey, the online questionnaire took 5-10 min to finish. Follow-up surveys mainly collected the following information: (1) current jobs: working location, professional title, job income, formally funded positions, especially for CSP graduates etc.; (2) career mobility: whether change jobs and reasons, and information about new jobs etc.; and (3) career development: promotion, residency training, and job performance etc. The content of each follow-up survey was similar. Whether breaking the contracts for CSP graduates and job income for both types of graduates could be invasive. However, these questions were asked repeatedly in each follow-up survey, the content of follow-up surveys was unlikely to have a differential impact on attrition.

### Strategies for increasing response

To maintain the participation of medical graduates in the cohort, we mainly adopted the following strategies. First, in between scheduled follow-ups, regular newsletters were sent to the WeChat groups by project members, including national or regional policies related to CSP, newly published research concerning CSP, and greetings on important holidays. Second, we organized a writing competition each year to encourage graduates who have become GPs in rural and remote areas to share their experiences. Third, we built a liaison team composed of student leaders in the baseline survey, and asked them to send the questionnaire to graduates who did not respond.

To increase response rate, we offered ten CNY as allowances for those who completed questionnaires. If participants did not fill in the questionnaire in time, we would add them as WeChat friends, call them, or send E-mails to remind them. Besides, we would also ask liaison members to contact and remind them to fill in the questionnaire.

### Measurement

#### 1. Follow-up status

Participants were categorized into two mutually exclusive groups: (1) complete follow-up refers to those who finished all follow-up surveys; (2) incomplete follow-up refers to those who did not finish at least one follow-up survey.

Participants with incomplete follow-up were further categorized into three mutually exclusive sub-groups: (1) “always-out” refers to those who did not respond to any follow-up surveys after the baseline visit, and those who could no longer be contacted through their phone, WeChat, E-mail, or other contact ways; (3) “rejoin” refers to those who finished the 2020 follow-up survey but was lost at least once in previous follow-up surveys; (4) “other” refers to those who did not respond to the 2020 follow-up survey but finished at least one previous follow-up surveys.

#### 2. Predictors of attrition and outcome

For potential predictors of attrition, we tried to cover variables that were found to have impact on attrition in previous literature, and also explored some variables related to medical study. The variables were categorized into sociodemographic characteristics [18, 19], attitudes towards medical study, preferences towards career development [20], and response behaviors in the baseline survey [21]. Sociodemographic information included types of students (whether CSP or NCSP graduate), medical university (Qinghai, Guangxi, Jiujiang, Gannan), graduation year (2015-2019), gender, family background (rural or urban) [22], highest completed education level of theirs parents (low middle school and below, or high middle school and above) [18], occupation of father (farmer or non-farmer), pressure from tuition and other fee at school (having pressure or no pressure), and household members (four family members or above, three family members or below) [19]. Attitudes towards medical study comprised of whether studying medicine was the first choice, whether planning to pursue a postgraduate degree, whether willing to participate in residency training, whether satisfied with medical education received, and whether understanding compulsory services program policy for CSP graduates.

Preferences of career development included whether desired to work in public hospitals above county level after graduation, whether believing they can pass the China National Medical Licensing Examination within one year, whether income was the primary consideration when looking for jobs, and whether the contract-signing place was hometown for CSP graduates.

Response behaviors included whether provide the score of university entrance examination, whether provide contact information (we collected WeChat, QQ, E-mail, and cellphone, providing at least one way of contact was considered “provide”. Providing none of the aforementioned contact was considered “not provide”). It should be noted that although we established WeChat groups in the baseline survey, students can still withdraw anytime from the group. Whether reporting household income was also included as a predictor of response behavior.

### Statistical analysis

To answer question 1, descriptive analyses were used to present the response rate of five sub-cohorts for each follow-up survey and the cumulative follow-up status ratio.

To answer question 2, firstly, the baseline characteristics between complete and incomplete follow-up of medical graduates, and among three types of attrition groups were presented. Chi-square tests were used to compare the differences between different groups of follow-up status. The P-value below 0.05 was considered statistically significant. Secondly, logistic regression was performed to investigate the factors associated with attrition of the cohort (the dependent variable was 1 for completing all follow-up surveys and 0 for incomplete) in the total sample and by types of graduates. Demographic characteristics, attitudes towards medical study, preferences towards career development, and response behaviors in baseline were included as explanatory factors in the model. In order to examine the predictors associated with rejoining, we conducted logistic regression in the incomplete sample (the dependent variable was 1 for rejoin and 0 for always-out or other). Thirdly, to rule out the effect of time, the Anderson and Gill model (AG model) was used to conduct a time-to-event analysis with recurrent events [23]. The detailed process and results was presented in the additional file.

To answer question 3, we adopted a multiple imputation method which has been widely used in dealing with missing data [24]. We performed a multiple imputation with chained equations (MICE), and all baseline variables were used to impute the interested outcome measures. For outcome measures, we chose three indicators that can reflect the career development of medical graduates, including income of the participant’s current job in the 2020 wave (income was collected by the question: “your income of the current job was___CNY per month”), whether passing the China National Medical Licensing Examination (NMLE) till 2020 wave, and obtaining a professional promotion till 2020 wave. The package “ice” was used to conduct the multiple imputation process in Stata.[25]. For the three outcome measures, comparisons were made between the imputed data and the data before imputation. T-tests were used for job income, and Chi-square tests were used for the other two outcome measures. Stata 16.0 (Stata Corp LP, College Station, TX, USA) and R version 4.0.2 were used to perform the analysis.

## Results

In total, 3620 medical graduates were included in the baseline survey from 2015 to 2019. There were 620 participants in the 2015 sub-cohort, 739 in 2016, 811 in 2017, 749 in 2018, and 701 in 2019. The numbers were similar in CSP and NCSP medical graduates.

### Attrition status in follow-up surveys

Table 1 describes the response rate of the study cohort in different follow-up surveys. In the first follow-up survey, the response rate was generally high. In the 2015 sub-cohort, the response rate was 91.9% in total, 91.5% for CSP graduates, and 92.4% for NCSP graduates. The response rate declined in each follow-up survey. After three follow-up surveys, the response rate was only 67.7%. The response rate was relatively higher in CSP graduates (78.4%) than that in NCSP graduates (57.5%) in the fourth follow-up survey.

**Table 1:** The response rate of the sub-cohorts by follow-up surveys, stratified by types of graduates N(%)

Follow-up surveys	Sub-cohorts				
	2015	2016	2017	2018	2019
—— All ——					
Baseline N	620 (100%)	739 (100%)	811 (100%)	749 (100%)	701 (100%)
First	570 (91.9%)	577 (78.1%)	585 (72.1%)	529 (70.6%)	612 (87.3%)
Second	424 (68.4%)	592 (80.1%)	586 (72.3%)	NA	NA
Third	474 (76.5%)	493 (66.7%)	NA	NA	NA
Fourth	420 (67.7%)	NA	NA	NA	NA
—— CSP ——					
Baseline N	305 (100%)	437 (100%)	481 (100%)	437 (100%)	381 (100%)
First	279 (91.5%)	373 (85.4%)	352 (73.2%)	328 (75.1%)	351 (92.1%)
Second	277 (90.8%)	383 (87.6%)	383 (79.6%)	NA	NA
Third	298 (97.7%)	330 (75.5%)	NA	NA	NA
Fourth	239 (78.4%)	NA	NA	NA	NA
—— NCSP ——					
Baseline N	315 (100%)	302 (100%)	330 (100%)	312 (100%)	320 (100%)
First	291 (92.4%)	204 (67.6%)	233 (70.6%)	201 (64.4%)	261 (81.6%)
Second	181 (46.7%)	209 (69.2%)	203 (61.5%)	NA	NA
Third	176 (55.9%)	163 (54.0%)	NA	NA	NA
Fourth	181 (57.5%)	NA	NA	NA	NA

Notes: *NA* not applicable

**Fig. 2:**
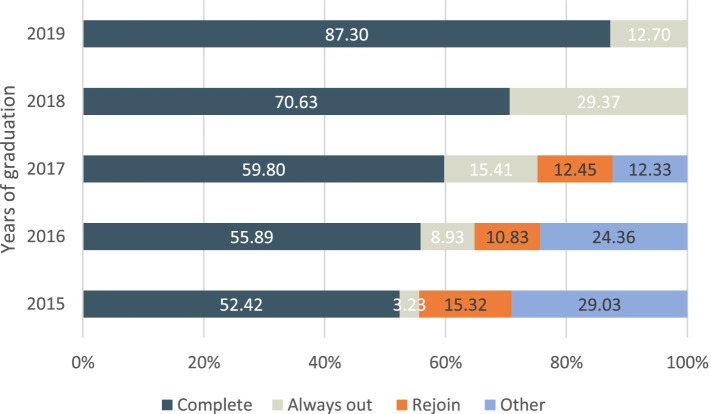
Cumulative follow-up status ratio in 2015-2019 sub-cohorts

Figure 2 shows the cumulative follow-up status ratio by pooling all follow-ups together. Despite differences across five sub-cohorts, we found a sizable proportion of attritors who have at least one lost-to-follow-up rejoined in the 2020 follow-up survey. In the 2015, 2016 and 2017 graduates, 15.3%, 10.8% and 12.5% rejoined in the 2020 follow-up survey, respectively. In the 2018 and 2019 graduates, because only one follow-up survey was conducted, by definition there were no ‘rejoin’ or ‘other’ categories.

### Determinants of attrition: baseline characteristics by attrition status

Table 2 presents the baseline characteristics of demographic information, attitudes towards medical study, preferences of career development, and response behaviors. Participants with complete follow-up and those with incomplete follow-up showed significant differences. For demographic characteristics, participants with incomplete follow-up were more likely to have or to be NCSP graduates (*P*<0.001), urban background (*P*=0.001), non-farmer father (*P*<0.001), having tuition or fee pressure during school (*P*<0.001), coming from a smaller family (three family members and below, *P*<0.001), and having better-educated parents (high middle school or above, *P*=0.012). For attitudes towards medical study, participants with incomplete follow-up were more likely to choose to study medicine as their first choice when choosing college majors (*P*=0.02), plan to pursue postgraduate study (*P*=0.004), would like to participate in residency training (*P*=0.001), show unsatisfied feelings towards undergraduate medical education (*P*<0.001). As for preferences of career development, participants with incomplete follow-up tended to be less confident of oneself in passing the NMLE within one year (*P*=0.002). CSP graduates with incomplete follow-up were more likely to sign contracts with hometowns (*P*<0.001). About response behaviors, very few graduates did not provide contact information (since we asked them to provide several ways of contact), but the proportion of not providing contact was higher in the incomplete group (7.6% in incomplete group, 2.9% in complete group, *P*<0.001). The proportion of not providing the university entrance exam score, not reporting household income were both higher in the incomplete group (Table 2).

**Table 2:** Distribution of baseline characteristics, stratified by follow-up status and groups of attrition

Characteristics	Follow-up status			Attrition by groups (vs. complete)		
	Complete N (column %)	Incomplete N (column %)	*P*-value	Always-out N (column %)	Rejoin N (column %)	Other N (column %)
**Demographic information**	2364	1256		520	276	460
CSP	1495 (63.2%)	546 (43.5%)	<0.001	214 (41.2%) ***	136 (49.3%) ***	196 (42.6%) ***
Medical schools						
Qinghai	689 (29.1%)	455 (36.2%)	<0.001	205 (39.4%) ***	71 (25.7%)	179 (38.9%) ***
Guangxi	719 (30.4%)	214 (17.0%)	<0.001	80 (15.4%) ***	32 (11.6%) ***	102 (22.2%) ***
Jiujiang	283 (12.0%)	197 (15.7%)	0.002	65 (12.5%)	73 (26.4%) ***	59 (12.8%)
Gannan	673 (28.5%)	390 (31.1%)	0.104	170 (32.7%)	100 (36.2%) **	120 (26.1%)
Male	1186 (50.2%)	643 (51.5%)	0.454	252 (49.2%)	152 (55.1%)	239 (52.0%)
Rural background	1584 (67.3%)	769 (61.8%)	0.001	294 (57.6%) ***	182 (66.2%)	293 (63.7%)
Highest education level for parents were low middle school and below	1431 (60.7%)	703 (56.3%)	0.012	291 (56.8%)	153 (55.4%)	259 (56.3%)
Father’s occupation=farmer	1260 (53.7%)	544 (43.7%)	<0.001	231 (45.3%) ***	122 (44.4%) **	191 (41.6%) ***
Enduring pressure from tuition and other fee during school	2157 (93.1%)	1082 (89.4%)	<0.001	436 (87.4%) ***	237 (89.8%)	409 (91.5%)
Four family members and above	955 (40.5%)	417 (33.5%)	<0.001	154 (30.2%) ***	87 (31.5%) **	176 (38.3%)
**Attitudes towards medical study**						
Studying medicine was first choice	1817 (77.4%)	919 (73.9%)	0.020	375 (73.7%)	203 (73.8%)	341 (74.1%)
Planning to pursue postgraduate study	1439 (61.2%)	818 (66.1%)	0.004	354 (69.8%) ***	167 (60.7%)	297 (65.3%)
Willing to participate in residency training	1957 (83.3%)	904 (73.0%)	<0.001	374 (73.2%) ***	201 (73.1%) ***	329 (72.6%) ***
Satisfied with medical education received	1188 (50.3%)	540 (43.4%)	<0.001	235 (46.1%)	112 (40.9%) **	193 (42.0%) **
Understanding compulsory services program	1385 (92.7%)	499 (91.4%)	0.323	195 (91.1%)	122 (89.7%)	182 (92.9%)
**Preferences of career development**						
Desired to work in public hospitals above county level after graduation	1554 (65.8%)	844 (67.7%)	0.233	368 (72.2%) **	171 (62.0%)	305 (66.3%)
Confident of passing NMLE within one year	1200 (52.2%)	562 (46.7%)	0.002	250 (50.3%)	134 (50.0%)	178 (40.6%) ***
Income was the primary consideration when applying for jobs	853 (36.2%)	400 (32.2%)	0.017	187 (36.7%)	82 (29.8%)	131 (28.5%) **
Contract-signing place was hometown	906 (63.4%)	276 (53.8%)	<0.001	103 (52.0%)	75 (59.1%)	98 (52.1%) **
**Response behaviors in baseline**						
Providing scores for university entrance exam	1575 (67.0%)	775 (62.7%)	0.009	279 (54.8%) ***	191 (70.0%)	305 (67.0%)
Providing contact information	2296 (97.1%)	1160 (92.4%)	<0.001	465 (89.4%) ***	256 (92.8%) ***	439 (95.4%) *
Reporting household income	2250 (95.2%)	1133 (90.2%)	<0.001	468 (90.0%) ***	252 (91.3%) **	413 (89.8%) ***

Notes: (1) Source: Compulsory Services Program 2015-2020 cohort; (2) Chi-square test was used for categorical variables, with p-value reported. *** *p*<0.001, ** *p*<0.05, * *p*<0.01 (3) “Contract-signing place was hometown” and “Understanding compulsory services program” are only for CSP graduates. (4) NMLE, China National Medical Licensing Examination; NA, not applicable

The baseline characteristic differences with the complete group were similar with those who rejoin and those who partially respond (“other”). However, the always-out group seemed to differ more significantly from the complete group. In the always-out group, the proportion of participants with a rural background, father’s occupation being farmer, having tuition pressure, having three family members and below were significantly smaller than that in the complete group (all *P*<0.001) (Table 2).

### Factors associated with attrition

Table 3 describes the factors associated with complete follow-up and rejoin estimating by multivariable logistic regressions. The total sample was used in regression (1), and the dependent variable was whether complete follow-up surveys (1=incomplete, 0=complete). CSP graduates (OR=0.40, 95%CI[0.33 - 0.48]), and having four family members and above (OR=0.84, 95%CI[0.71 - 1.00]) predicted lower incomplete follow-up. Graduates who chose to study medicine as their first choice (OR=0.78, 95%CI[0.65 - 0.95]), and those who were willing to participate in residency training (OR=0.80, 95%CI[0.66 - 0.98]) were associated with a lower possibility of incomplete follow-up. Providing scores for the university entrance exam (OR=0.82, 95%CI[0.69 - 0.97]), providing contact information (OR=0.46, 95%CI[0.32 - 0.66]) and providing household income (OR=0.60, 95%CI[0.43 - 0.84]) all significantly reflected the lower possibility of incomplete follow-up (Table 3).

**Table 3:** Baseline factors associated with incomplete follow-up and rejoining: multivariable logistic regression

Variables	(1)		(2)		(3)	
	All sample		Graduates of 2015-2017		Incomplete samples	
	OR	95% CI	OR	95% CI	OR	95% CI
**Demographic information**						
Types of students (0=NCSP)	0.40***	(0.33 - 0.48)	0.37***	(0.29 - 0.46)	1.52**	(1.05 - 2.19)
Male (0=female)	1.07	(0.90 - 1.25)	1.13	(0.93 - 1.39)	0.99	(0.72 - 1.37)
Rural background (0=urban)	0.97	(0.80 - 1.18)	0.99	(0.78 - 1.26)	1.26	(0.86 - 1.85)
Highest education level of parents (0=low middle school and below)	1.02	(0.85 - 1.22)	1.00	(0.80 - 1.24)	1.19	(0.84 - 1.70)
Occupation of father (0=not farmer)	0.89	(0.74 - 1.08)	0.83	(0.66 - 1.04)	1.33	(0.91 - 1.94)
Pressure from tuition and other fee during school (0=no)	0.76	(0.57 - 1.01)	0.65**	(0.45 - 0.95)	0.97	(0.57 - 1.65)
Household size (0=3 and below)	0.84**	(0.71 - 1.00)	0.90	(0.73 - 1.11)	0.83	(0.58 - 1.17)
**Attitudes towards medical study**						
Studying medicine was first choice (0=no)	0.78**	(0.65 - 0.95)	0.81	(0.64 - 1.01)	1.13	(0.79 - 1.62)
Planning to pursue postgraduate study(0=no)	0.90	(0.75 - 1.08)	0.86	(0.69 - 1.08)	0.83	(0.58 - 1.19)
Willing to participate in residency training(0=no)	0.80**	(0.66 - 0.98)	0.76**	(0.60 - 0.97)	0.93	(0.64 - 1.36)
Satisfied with medical education received(0=no)	0.95	(0.81 - 1.12)	0.99	(0.81 - 1.21)	0.91	(0.65 - 1.26)
Understanding compulsory services program (0=no)	-	-	-	-	-	-
**Preferences of career development**						
Desired to work in public hospitals above county level after graduation (0=no)	1.00	(0.84 - 1.20)	1.06	(0.85 - 1.31)	0.89	(0.63 - 1.25)
Confidence of passing NMLE within one year (0=no)	0.99	(0.84 - 1.16)	1.03	(0.84 - 1.26)	1.14	(0.82 - 1.59)
Income was the primary consideration when applying for jobs (0=no)	1.10	(0.93 - 1.31)	1.08	(0.87 - 1.34)	0.89	(0.63 - 1.26)
Contract-signing place was hometown (0=no)	-	-	-	-	-	-
**Response behaviors in baseline**						
Providing scores for university entrance exam (0=no)	0.82**	(0.69 - 0.97)	0.76**	(0.62 - 0.94)	1.64***	(1.15 - 2.33)
Providing contact information (0=no)	0.46***	(0.32 - 0.66)	0.36***	(0.22 - 0.60)	0.82	(0.43 - 1.54)
Providing household income (0=no)	0.60***	(0.43 - 0.84)	0.60**	(0.40 - 0.89)	0.87	(0.49 - 1.54)
Constant	17.33***	(8.89 - 33.81)	27.90***	(12.00 - 64.87)	0.22***	(0.07 - 0.65)
Number of observations	3,310		1,930		840	

Notes: (1) Source: Compulsory Services Program 2015-2020 wave; (2) Robust 95% CI in parentheses, *** *p*<0.01, ** *p*<0.05 (3) Logistic regression was conducted, with odds ratio reported. The schools and years of graduation for each sub-cohort were controlled for in all regressions to control for cohort effects. (4) The dependent variable was dichotomous, 1=incomplete and 0=complete; in regression 3, the incomplete samples included always-out, rejoin, and other, and the dependent variable was 1 for rejoin and 0=always-out or other. (5) *NMLE* China National Medical Licensing Examination; *OR* odds ratio; *CI* confidence interval; *NA* not applicable; *NCSP* non-compulsory services program

In regression (2), we restricted the sample to graduates of 2015-2017, excluding graduates of 2018 & 2019 who had only one follow-up survey. The results were similar to that in regression (1). However, the tuition/fee pressure in school was associated with a lower possibility of incomplete follow-up surveys (OR=0.65, 95%CI[0.45 - 0.95]). Wiliness of studying medicine was not significant anymore. Overall, these two factors might have an inconsistent effect on follow-up status (Table 3). We further conducted separate estimates for CSP and NCSP graduates, the results were reported in the additional file.

### Factors associated with rejoining in the survey

In order to explore the factors associated with rejoining, we conducted Logistic regression among the attrition participants (Table 3). CSP graduates were 1.52 times more likely to come back to the survey than NCSP graduates (P<0.05). Medical graduates who provided university entrance exam scores were 1.64 times more likely to rejoin in the 2020 follow-up survey compared with those who did not provide (P<0.05).

### Estimating the effect of attrition on three outcome measures

In Table 4, we compared the data before imputation and imputed data for three key outcome measures related to career development (Table 4). Overall, the results showed that the three imputed outcome measures did not have significant difference with the outcome measures before imputation, which indicated that attrition might have no impact on outcomes. For job income, before imputation, the mean job income was 4022.8 CNY for CSP graduates, and 4065.3 CNY after imputation. The imputed job income did not have significant differences compared with that before imputation (P=0.104). for whether passing the NMLE, 93.8% and 93.3% of CSP graduates passed the NMLE before and after imputation, respectively, and the imputed data did not differ significantly with that before imputation (P=0.148). for whether obtaining a professional title, there were 62.0% of CSP graduates obtained a title in the dataset; after imputation, 61.7% of CSP graduates obtained a title, and the difference was not significant, again. The results were similar for NCSP graduates.

**Table 4:** Results of three outcome variables of career development before and after multiple imputation

Variables	CSP (N=1233)			NCSP (N=947)		
	Before imputation	After imputation	*P*-value^#^	Before imputation	After imputation	*P*-value^#^
Income of current job	4022.8 (3868.9)	4065.3 (3555.9)	0.104	6456.1 (5267.2)	6420.9 (4269.9)	0.714
Missing	301	36		547	61	
Whether passing the NMLE						
No	59 (6.2%)	80 (6.7%)	0.148	51 (9.3%)	91 (10.1%)	0.329
Yes	893 (93.8%)	1110 (93.3%)		496 (90.7%)	809 (89.9%)	
Missing	271	33		400	47	
Whether obtained a professional title						
No	362 (38.0%)	456 (38.3%)	0.676	322 (58.9%)	527 (58.6%)	0.814
Yes	590 (62.0%)	734 (61.7%)		225 (41.1%)	373 (41.4%)	
Missing	271	33		400	47	

Notes: (1) Source: Compulsory Services Program 2015-2017 wave; (2) Mean and standard deviation are reported for income; N and column % are reported for categorical variables; (3) The P-value was obtained by comparing data before imputation and the imputed data, not with the total data after imputation. T-test was used to examine the differences of “income of current job”, and Chi-square test was used for “whether passing the NMLE” and “whether obtained a professional title”; (4) *NMLE* China National Medical Licensing Examination; *CSP* compulsory services program, *NCSP* non-compulsory services program

## Discussion

This paper examined the determinants of attrition in a prospective cohort comprised of young medical graduates, explored factors associated with attrition and rejoining, and assessed the impact of attrition on outcome measures of career development for young medical graduates. Several key findings were highlighted. First, the attrition increased with more follow-up surveys and 67.7% of the participants remained in the cohort after four follow-up surveys. The cumulative follow-up ratio was above 50% in all five sub-cohorts. Second, graduates who reported lower preference towards residency training and who did not provide sensitive information were associated with higher attrition, while CSP graduates and those who provided university entrance scores were more likely to rejoin the cohort. Third, despite the systematic differences detected among different follow-up status, no statistically significant impacts were found on the outcome measures.

In countries that established similar compulsory services policies or programs aiming to bridge the health workforce gap, we did not find studies that examine attrition in similar cohorts of medical graduates [26]. Japan established a CSP in 1972 in Jichi Medical University and trained rural doctors under contracts. However, the study they conducted was a retrospective cohort study rather than a prospective one [27, 28]. Graduates with certain characteristics may be reluctant to join in the study, which would cause selection bias, decreasing the power of the study. Among other cohort studies that focused on health professionals, the MABEL longitudinal survey in Australia was a famous large nationwide prospective cohort study, aiming to investigate factors influencing participation and supply of health workforce, specialty choice and mobility of health workforce. They included new doctors in subsequent follow-up to replace any attrition and maintain the cross-sectional representativeness of each follow-up [8, 29], so their patterns of attrition would be systematically different from ours.

CSP had a significant higher response rate compared with NCSP graduates. There are several potential explanations. CSP students need to sign contracts with medical universities and local governments and promise to practice in designated rural areas for six years. Through fulfilling the contracts, CSP graduates’ spirits of contract and sense of responsibility are intensified virtually. They are more likely to remain in the cohort and fill in the follow-up questionnaires. After several follow-up surveys, participation might become a habit so that participants no longer consider whether to respond or not, but “participate because they have done so all along” [30]. For NCSP graduates, they do not possess a contract or even any obligations of becoming doctors. NCSP graduates are more likely to leave the medical industry and pursue other careers than CSP graduates. Choosing careers elsewhere indicates that medicine is not their interests, and surveys related to medical career development become less attractive to them.

Unwillingness to participate in residency training was associated with a higher possibility of attrition. According to the national policy in China starting in 2015, all medical graduates need to participate in residency training after graduation [14], which generally lasts for three years [31]. For CSP graduates, the three years of training are included in the six-year service period stipulated in the contract. Not willing to participate in residency training suggests that the CSP graduates may be more likely to break the contracts and the NCSP graduates are more likely to leave the medical industry, so they would be reluctant to remain in the cohort established in their medical universities.

We found the response behaviors were consistently associated with attrition. Graduates who did not provide scores for the university entrance exam, contact information, or household income in the baseline survey were more likely to attrite. This finding was consistent with previous research [22, 32]. The completeness of information provided can reflect participants’ willingness towards the survey and persistently participating in the survey. Not providing contact information could be viewed as an indirect way of withdrawing from the cohort study or a ‘polite’ refusal. Students who did not provide key information at baseline should be targeted early since they are ready to leave cohorts from the beginning.

More than 10% of rejoining participants indicates that those rejoiners should be examined for scrutiny [33]. CSP and reporting score for university entrance exam predicted higher possibility of rejoining. Cheng and Trivedi (2015) estimated the attrition bias in the MABEL study [3]. They also found a significant faction of attriting physicians (approximately 23 to 32% of the drop-outs) rejoined the study. They attributed this phenomenon to changes in work (or residential) address, causing them unreachable. Since our follow-up surveys are conducted online, we think there are other two factors contributing to rejoining. First, the work status may help to explain the rejoining. Graduates need time to fit in the new working environment in their new occupations. This period of adaption poses more challenges on them, resulting in no time or interest to handle a non-mandated survey. After settling down in the new environment, they would have more control over their time so they would fill in the follow-up questionnaires. Besides, the work attitudes and efforts of investigators may also explain the rejoining to some extent.

Importantly, the systematic differences across attrition groups seemed not to have significant impact on key outcome measures of career development. Our finding adds to a growing body of literature suggesting that selective attrition does not necessarily twist the estimates of outcomes [34–36]. It might be optimistic to interpret future results without special caution on attrition bias based on current response rate.

A key strength of this study was that we examined the attrition and rejoin in a prospective cohort comprised of young medical graduates. Younger population and medical professionals are less likely to stay in the cohort as aforementioned, but we managed to assess attrition in a cohort composed of young medical graduates. To our knowledge, this is the first study examining attrition in such a cohort. A limitation of this study is the short follow-up period. The first group of graduates were only followed four times, and graduates of 2018 and 2019 were only followed once due to COVID-19 pandemic. It particularity limited the sample size when assessing the impact of attrition on outcome measures of career development. The career development of those graduates remains to be observed in the future. Another limitation is unmeasured confounders that would potentially affect follow-up status and outcome measures, for example, the working mode, or the professionalism of the graduates. Future studies should take those unmeasured factors into consideration when interpreting results.

## Conclusions

In a prospective cohort comprised of young health professionals, those who were unwilling to participate in residency training and those who did not provide sensitive information were associated with higher attrition. These graduates should be targeted early. The researchers should provide more background information of the study to help them understand the meaning of their participation. For graduates who have the potential of rejoining, more efforts should be invested to maintain their participation. Despite the systematic differences detected among different follow-up status, they do not have significant impacts on the outcome measures of career development.

## Supplementary Information

**Additional file 1.**

## Data Availability

The datasets generated and/or analyzed during the current study are not publicly available due to limitations of ethical approval involving the personal data and anonymity but are available from the corresponding author on reasonable request.
