# Supplementary material for: Sample attrition analysis in a prospective cohort study of medical graduates in China
Source: BMC Med Res Methodol. 2022 Jan 14;22:14. doi: 10.1186/s12874-021-01498-1 (PMC8758240; doi:10.1186/s12874-021-01498-1)
Supplement: Supplementary file 1 — Additional file 1. [file 12874_2021_1498_MOESM1_ESM.docx]

**Sample attrition analysis in a prospective cohort study of medical graduates in China**

**Additional file**

**Table s1.** Baseline factors associated with incomplete follow-up and rejoining stratified by CSP and NCSP: multivariable logistic regression

| Variables | Graduates of CSP | | Graduates of NCSP | |
| --- | --- | --- | --- | --- |
|  | OR | 95% CI | OR | 95% CI |
| **Demographic information** |  |  |  |  |
| Male (0=female) | 1.21 | (0.96 - 1.53) | 0.90 | (0.70 - 1.14) |
| Rural background (0=urban) | 0.91 | (0.68 - 1.22) | 1.01 | (0.77 - 1.33) |
| Highest education level for parents (0=low middle school and below) | 1.02 | (0.79 - 1.32) | 1.08 | (0.83 - 1.39) |
| Occupation of father (0=not farmer) | 0.82 | (0.62 - 1.08) | 0.96 | (0.73 - 1.26) |
| Pressure from tuition and other fee during school (0=no) | 0.95 | (0.60 - 1.48) | 0.63** | (0.42 - 0.95) |
| Household size (0=3 and below) | 0.76** | (0.59 - 0.97) | 0.96 | (0.75 - 1.24) |
| **Attitudes towards medical study** |  |  |  |  |
| Studying medicine was first choice (0=no) | 0.84 | (0.65 - 1.09) | 0.82 | (0.60 - 1.12) |
| Planning to pursue postgraduate study(0=no) | 1.00 | (0.79 - 1.26) | 0.69** | (0.51 - 0.94) |
| Willing to participate in residency training(0=no) | 0.58*** | (0.42 - 0.82) | 0.87 | (0.67 - 1.12) |
| Satisfied with medical education received(0=no) | 0.81* | (0.64 - 1.02) | 1.23* | (0.97 - 1.57) |
| Understanding compulsory services program (0=no) | 0.80 | (0.52 - 1.22) | NA | NA |
| **Career development** |  |  |  |  |
| Desired to work in public hospitals above county level after graduation (0=no) | 1.13 | (0.89 - 1.43) | 0.84 | (0.61 - 1.15) |
| Confidence of passing NMLE within one year (0=no) | 0.87 | (0.69 - 1.12) | 1.10 | (0.87 - 1.40) |
| Income was the primary consideration when applying for jobs (0=no) | 0.92 | (0.72 - 1.17) | 1.34** | (1.03 - 1.75) |
| Contract-signing place was hometown (0=no) | 0.88 | (0.67 - 1.15) | NA | NA |
| **Responses to questionnaires** |  |  |  |  |
| Providing scores for university entrance exam (0=no) | 0.76** | (0.59 - 0.98) | 0.82 | (0.64 - 1.05) |
| Providing contact information (0=no) | 0.51*** | (0.31 - 0.85) | 0.38*** | (0.21 - 0.70) |
| Providing household income (0=no) | 0.72 | (0.40 - 1.29) | 0.60** | (0.39 - 0.92) |
| Constant | 5.59*** | (1.96 - 15.96) | 32.17*** | (11.96 - 86.51) |
| Number of observations | 1,754 |  | 1,468 |  |

Notes: (1) Source: Compulsory Services Program 2015-2020 wave; (2) Robust 95% CI in parentheses, *** p<0.01, ** p<0.05 (3) Logistic regression was conducted, with odds ratio reported. The four schools and the year of onset of the five cohorts were controlled for in all regressions. (4) The dependent variable was dichotomous, 1=incomplete and 0=complete; (5) NMLE, China National Medical Licensing Examination; OR, odds ratio. CI, confidence interval; NA, not applicable; NCSP: non-compulsory services program.

**Table s2**. Demographic characteristics of the five sub-cohorts

| Characteristics | 2015 | 2016 | 2017 | 2018 | 2019 |
| --- | --- | --- | --- | --- | --- |
|  | N=620 | N=739 | N=811 | N=749 | N=701 |
| Types of students |  |  |  |  |  |
| NCSP | 315 (50.8%) | 302 (40.9%) | 330 (40.7%) | 312 (41.7%) | 320 (45.6%) |
| CSP | 305 (49.2%) | 437 (59.1%) | 481 (59.3%) | 437 (58.3%) | 381 (54.4%) |
| School |  |  |  |  |  |
| Qinghai | 162 (26.1%) | 256 (34.6%) | 216 (26.6%) | 253 (33.8%) | 257 (36.7%) |
| Guangxi | 171 (27.6%) | 185 (25.0%) | 190 (23.4%) | 192 (25.6%) | 195 (27.8%) |
| Jiujiang | 146 (23.5%) | 140 (18.9%) | 75 (9.2%) | 63 (8.4%) | 56 (8.0%) |
| Gannan | 141 (22.7%) | 158 (21.4%) | 330 (40.7%) | 241 (32.2%) | 193 (27.5%) |
| Gender |  |  |  |  |  |
| Female | 297 (47.9%) | 356 (48.2%) | 373 (46.2%) | 392 (52.8%) | 363 (51.8%) |
| Male | 323 (52.1%) | 383 (51.8%) | 434 (53.8%) | 351 (47.2%) | 338 (48.2%) |
| Background |  |  |  |  |  |
| Urban | 189 (30.5%) | 253 (34.3%) | 268 (33.5%) | 287 (38.6%) | 250 (35.7%) |
| Rural | 430 (69.5%) | 484 (65.7%) | 533 (66.5%) | 456 (61.4%) | 450 (64.3%) |
| Highest education level for parents |  |  |  |  |  |
| Low middle school and below | 375 (60.6%) | 404 (54.8%) | 472 (58.6%) | 427 (57.3%) | 456 (65.1%) |
| High middle school and above | 244 (39.4%) | 333 (45.2%) | 334 (41.4%) | 318 (42.7%) | 244 (34.9%) |
| Occupation of father |  |  |  |  |  |
| Not farmer | 370 (60.4%) | 342 (46.5%) | 388 (48.3%) | 370 (49.9%) | 315 (45.2%) |
| Farmer | 243 (39.6%) | 393 (53.5%) | 415 (51.7%) | 371 (50.1%) | 382 (54.8%) |
| Pressure from tuition and other fee during school |  |  |  |  |  |
| No | 38 (6.3%) | 57 (8.0%) | 64 (8.2%) | 70 (9.5%) | 60 (8.7%) |
| Yes | 568 (93.7%) | 657 (92.0%) | 718 (91.8%) | 663 (90.5%) | 633 (91.3%) |
| Household size |  |  |  |  |  |
| Three and below | 366 (59.1%) | 456 (62.0%) | 506 (62.9%) | 476 (63.9%) | 428 (61.2%) |
| Four and above | 253 (40.9%) | 280 (38.0%) | 299 (37.1%) | 269 (36.1%) | 271 (38.8%) |

The Anderson and Gill model (AG model) assumes that the correlation between event times for a person can be explained by past events, and it has been used to evaluate repeated occurrence of basal cell carcinoma and recurrent hospitalizations (Amorim & Cai, 2015). For each follow-up survey, not filling in the survey was considered the “event”, and time was measured in months. R version 4.0.2 was used to perform the analysis. The package “tmerge” in R was used to construct the dataset, and the library survival in R was used to fit the model. The results was presented in Table s3.

**Table s3.** Results of respondent characteristics on overall follow-up response: the Anderson and Gill model for recurrent events

| Variables | HR | P-value | 95% CI |
| --- | --- | --- | --- |
| **Demographic information** |  |  |  |
| Types of students (0=NCSP) | 0.46*** | <0.001 | (0.40-0.53) |
| Male (0=female) | 1.08 | 0.198 | (0.96-1.20) |
| Rural background (0=urban) | 0.97 | 0.615 | (0.85-1.10) |
| Highest education level for parents (0=low middle school and below) | 0.96 | 0.474 | (0.85-1.08) |
| Occupation of father (0=not farmer) | 0.93 | 0.290 | (0.82-1.06) |
| Pressure from tuition and other fee during school (0=no) | 0.76*** | 0.001 | (0.64-0.89) |
| Household size (0=3 and below) | 0.96 | 0.486 | (0.85-1.08) |
| **Attitudes towards medicine study** |  |  |  |
| Studying medicine was first choice (0=no) | 0.84*** | 0.004 | (0.74-0.94) |
| Planning to pursue postgraduate study(0=no) | 0.93 | 0.251 | (0.81-1.06) |
| Willing to participate in residency training(0=no) | 0.88* | 0.050 | (0.78-1.00) |
| Satisfied with medical education received(0=no) | 1.01 | 0.890 | (0.90-1.13) |
| **Career development** |  |  |  |
| Desired to work in public hospitals above county level after graduation (0=no) | 1.00 | 0.932 | (0.88-1.13) |
| Confidence of passing NMLE within one year (0=no) | 1.00 | 0.945 | (0.89-1.12) |
| Income was the primary consideration when applying for jobs (0=no) | 1.05 | 0.406 | (0.93-1.19) |
| **Responses to questionnaires** |  |  |  |
| Providing scores for university entrance exam (0=no) | 0.78*** | <0.001 | (0.70-0.87) |
| Providing contact information (0=no) | 0.62*** | <0.001 | (0.53-0.72) |
| Providing household income (0=no) | 0.86 | 0.053 | (0.73-1.00) |

Notes: (1) Source: Compulsory Services Program 2015-2017 wave; (2) Robust 95% CI in parentheses, ***p<0.01 *p<0.05 (3) Logistic regression was conducted, with hazard ratio reported. The schools and years of graduation for each sub-cohort were controlled for in the regression to control for cohort effects. (4) The dependent variable was dichotomous, 1=incomplete and 0=complete; (5) NMLE, China National Medical Licensing Examination; HR, hazard ratio. CI, confidence interval; NCSP: non-compulsory services program.
